# Gender Differences in Attention Adaptation after an 8-Week FIFA 11^+^ for Kids Training Program in Elementary School Children

**DOI:** 10.3390/children8090822

**Published:** 2021-09-18

**Authors:** Chia-Hui Chen, Ghazi Rekik, Yosra Belkhir, Ya-Ling Huang, Yung-Sheng Chen

**Affiliations:** 1 Sporting Education Office, National Taiwan University of Science and Technology, Taipei 10607, Taiwan; monkey0209@gmail.com; 2 Research Laboratory: Education, Motricity, Sport and Health, LR19JS01, Sfax University, Sfax 3000, Tunisia; ghazi.rek@gmail.com (G.R.); belkhir.ysr@gmail.com (Y.B.); 3 Tanyu Research Laboratory, Taipei 11266, Taiwan; 4 Al-Udhailiyah Primary School for Girls, Al-Farwaniyah 085700, Kuwait; 5 High Institute of Sport and Physical Education, Manouba University, Manouba 2010, Tunisia; 6 Sporting Education Office, Chang Gung University of Science and Technology, Taoyuan 33303, Taiwan; ylhuang@mail.cgust.edu.tw; 7 Department of Exercise and Health Sciences, University of Taipei, Taipei 111036, Taiwan; 8 Exercise and Health Promotion Association, New Taipei City 24156, Taiwan

**Keywords:** school-based exercise, pediatric health, concentration, gender difference, exercise intervention

## Abstract

School-based exercise intervention is recognized as an optimal tool for enhancing attentional performance in healthy school children. However, gender differences in the training adaptation regarding attentional capacities have not been elucidated clearly in the current literature. This study aimed to investigate the effects of an 8-week Fédération Internationale de Football Association (FIFA) 11^+^ for Kids training program on attentional performance in schoolboys and girls. Based on a quasi-experimental design, fifty-two children registered in year five of elementary school were assigned into the following groups: training boys (*n* = 13), training girls (*n* = 13), control boys (*n* = 13), and control girls (*n* = 13). The training groups undertook an 8-week FIFA 11^+^ Kids intervention with a training frequency of five times per week, whereas the control groups were deprived of any exercise during the study period. All the participants maintained their regular physical activity and weekly physical education (PE) lessons (two 50-min lessons per week of school curriculum) during the training period. The Chinese version of the Attention Scale for Elementary School Children (ASESC) test was used for attentional assessment at the baseline and one week after the interventional period. The Kruskal–Wallis H test was used for between-group comparison, whereas the Wilcoxon signed-rank test was used for within-group comparison. Significant differences in total scale, focused attention, selective attention, and alternating attention were found in group comparisons (*p* < 0.001). Furthermore, the training children significantly increased their values in relation to total scale, focused attention, sustained attention, and selective attention (*p* < 0.05). Only training girls significantly improved their divided attention after the training period (*p* < 0.001, MD = −0.77, ES = −0.12). In conclusion, the FIFA 11^+^ for Kids is an effective school-based exercise intervention for attentional improvement in school children. The schoolgirls demonstrated a positive outcome regarding divided attention after the interventional period.

## 1. Introduction

A fundamental aspect of school education is related to the attention and learning efficiency of students. Two questions that are always uppermost in all educators’ and parents’ minds are as follows: “Does this child have attention problems? How do I improve the attention span for children learning at school?” In the 1970s, Posner [1] stated that an attentional process involves multiple cognitive functions in the central nervous system. Later, a framework proposed by Posner and his colleagues [2] outlined a holistic representation of attention networks including alerting, orienting, and executive control aspects. Neurophysiological studies, particularly in neuroanatomic, neuroimaging and neurobiological evidence support the role of cerebral functions associated with attentional control [3]. For example, biogenetic studies have reported that biological factors, such as dopamine D4 receptor (DRD4), dopamine transporter (DAT1), catecholamine-O-methyl transferase (COMT), and monoamine oxidase (MAOA), are related to psychological behavior and attention functions [4,5].

Attentional modulation and plasticity in the human brain are evidenced by attentional training interventions and a proper learning environment in the early stages of growth [6,7]. During development, preadolescent boys display different characteristics regarding attentional performance in comparison to preadolescent girls; these differences are due to physiological, psychological, and social aspects. For example, Clarke et al. [8] reported that schoolboys showed fewer theta electroencephalography (EEG) brainwaves and more alpha EEG brainwaves than schoolgirls, indicating gender differences in brain function during childhood. Additionally, gender differences in attentional control have been found in children with attention deficit hyperactivity disorder (ADHD) (i.e., girls had lower Conners ADHD rating scales) [9]. It is well documented that daily physical activities and exercise interventions are strongly related to attentional performance in school-aged children [10,11,12,13]. In acute exercise intervention, a 12-min continuous running exercise can immediately improve selective attention in children [14]. Additionally, an acute bout of 20-min treadmill walking at a moderate intensity of 60% maximal heart rate results in functional improvement in attentional tasks in 10-year-old children [15]. The modulation of exercise-induced neurotransmitters in the cerebrum (such as adrenaline, dopamine, and brain-derived neurotrophic factors) is considered a primary mechanism to alter post-exercise attentional performance [16,17]. In terms of the chronic effects of exercise intervention, a longitudinal study reported that developing executive function skills before and during school age was strongly associated with elementary school mathematics performance, indicating the important role of attention and motor performance in the early development of school children [18]. Moreover, school-based exercise interventions can contribute to beneficial outcomes regarding attention control and academic performance in school-aged children [19]. Superior cognitive improvements and higher levels of physical engagement were also identified in school children who undertook a 6-week team game in comparison to those who undertook 6 weeks of aerobic exercise [20]. The cognitive benefits of chronic exercise interventions in children have been suggested as being a result of exercise-induced adaptation in cognitive–motor interactions of cerebral regions, such as the prefrontal cortex, motor cortex, and basal ganglia [21]. This assumption has been evidenced by an increase in spatial working memory after an 8-week gymnastics training program in school children [22].

The Fédération Internationale de Football Association (FIFA) 11^+^ for Kids is a structured training program with the aim of improving neuromuscular functions in preadolescent children [23,24]. Recent studies have reported on chronological adaptation in enhancing skill-related physical fitness components of youth football players [25,26,27]. For example, Rössler et al. [25] found that footballers aged 7–12 significantly improved their Y-balance capacity, jumping ability, agility, and dribbling ability after a 10-week FIFA 11^+^ for Kids program. However, the optimal benefits regarding the physiological adaptation of the FIFA 11^+^ for Kids were only reported in sports trained youth players. Recently, our laboratory [28] conducted an intensive 8-week FIFA 11^+^ for Kids training program in elementary school children. Our findings reported the positive benefits of physical fitness (e.g., sit-and-reach, broad jump, sit-up, and 800 m run) and attentional capacity. This finding implies that structure-based exercise interventions can positively improve attentional performance in school children.

To the best of our knowledge, information regarding the influence of gender differences in exercise training adaptations on attentional control has not yet been elucidated in the literature, particularly in relation to school children. Most importantly, we seek to identify which area of attentional performance can help schoolteachers deliver appropriate course curricula to approach the demands of individual children. Therefore, the purpose of this study was to compare gender differences in attentional adaptation after an 8-week FIFA 11^+^ for Kids training intervention in elementary school children. The secondary purpose of this study was to identify what attentional capacity could be adaptable to the exercise intervention. It was first hypothesized that the 8-week FIFA 11^+^ for Kids would enhance attentional capacities in training children. The second hypothesis was that training girls would be superior in terms of training adaptation in all attentional assessments, compared to training boys and control children.

## 2. Materials and Methods

### 2.1. Participants

**Figure 1:**
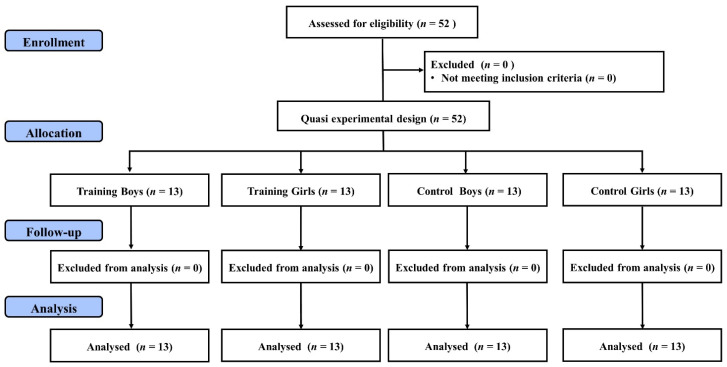
Experimental flow diagram of the study.

Fifty-two healthy children from a public elementary school voluntarily participated in this study (Sanchong district, New Taipei City, Taiwan). Based on a quasi-experimental design, thirteen children of the same gender were assigned into the FIFA 11^+^ for Kids Boys (training boys) group, the FIFA 11^+^ for Kids girls (training girls) group, control boys group, or control girls group (see Figure 1).

The inclusion criteria were as follows: (1) registered in the fifth year of elementary school; and (2) chronological age between 10–12. Exclusion criteria included the following: (1) current neurological or cardiovascular diseases; (2) psychological disorders; (3) taking medicine that affects psychometric status (e.g., benzodiazepines, anticonvulsants, antidepressants).

Prior to the experiment, all children and their parents and schoolteachers were informed of the scope of the study and the experimental procedure. All children were screened and there were no contraindications to participation. All children and parents signed informed consent forms. Subsequently, the children were familiarized with the experimental tests. This study was approved by the Human Research Ethics Committee of University of Taipei (UT-IRB-2020-003). This study was undertaken in accordance with the Declaration of Helsinki and its later amendments in 2013.

### 2.2. Experimental Procedure

The study was conducted during the spring semester of the school year. Based on the school curriculum and teaching schedule, one class of 26 children with an equal number of boys and girls was allocated to the training groups while another 13 boys and 13 girls from another class were assigned to the control groups. During the baseline stage, the participants undertook anthropometrics measurements for height (Seca 213; seca GmbH and Co. KG, Hamburg, Germany) and body weight (Xyfwt382; TECO, Taiwan) in the school health center. Afterwards, the participants performed the Chinese version of the Attention Scale for Elementary School Children (ASESC) test for attentional assessments in their classrooms. The participants were given hard copies of ASESC testing sheets and a pencil to complete the attentional assessment. The duration of the ASESC test was around 50 min in total (including resting intervals and ten tasks). The following week, the training groups began an 8-week FIFA 11^+^ kid intervention with a training frequency of five times per week. Conversely, participants in the control groups were deprived of any exercise intervention during the study period. All participants were told to maintain their regular physical activity and physical education (PE) lessons (two 50-minutee lessons per week of school curriculum) during the training period. A post-training ASESC test was conducted a week after the training period, following the same testing procedures as the baseline measurement. The participants were asked to refrain from strenuous exercises 24 h before the baseline and post-training assessments. A research assistant was blinded to conduct the ASESC tests in this study. One research fellow evaluated the score of attentional assessment in accordance with the ASESC test guidelines. Figure 1 shows the experimental flow of the study.

### 2.3. Training Intervention

The FIFA 11^+^ for Kids exercise program was used as a training intervention in this study. The program consisted of seven types of motor coordination exercises (running, skating jumps, single-leg stance, push-ups, single-leg jumps, spiderman, and sideways roll) with five variations (from basic to advance) [23]. Overall, a total of 35 exercises were included in the exercise program. The details of the FIFA 11^+^ for Kids intervention, as used in a school exercise program, are described in our recent publication [28].

During the training period, each training session (lasting 40-min) was conducted by a physical education teacher and a research assistant, both of whom were familiar with the FIFA 11^+^ for Kids program. The training boys and girls were instructed with appropriate movement operation and performance skills during training sessions. All training sessions started with a roll call at 8:00 a.m. on school days. All training boys and girls fully attended the training sessions during the training period.

### 2.4. Attentional Assessment

The Chinese version of the ASESC test developed by Lin and Chou [29] was used as an attentional assessment tool in this study. This scale is a reliable tool for a multi-dimensional attention test based on the “Clinical Attention Model” proposed by Sohlberg and Mateer [30,31]. The ASESC test consists of ten variants of attentional tasks (from item 1 to item 10) and is divided into (1) focused attention (item 1 and 2, 1 min for each test); (2) sustained attention (item 3 and 4, 5 min for each test); (3) selective attention (item 5 and 6, 1 min for each test); (4) alternating attention (item 7 and 8, 1 min for each test); and (5) divided attention (item 9 and 10, 2.5 min for each test).

Focused attention refers to an individual’s ability to directly respond to particular visual, auditory, or tactile stimuli. The subscale includes number-oriented and text-oriented subtests in which participants identify a specific number and Chinese characters. Sustained attention refers to an individual’s ability to maintain consistent behavioral responses during continuous and repetitive activities. This subscale includes petal comparison and digital circled subtests. Selective attention refers to an individual’s ability to maintain action and cognition in the presence of external stimuli or fierce competition. This subscale includes a map search and symbol recognition subtests. Alternating attention is the ability of an individual to control attentional allocations with the mental flexibility to switch between dissimilar cognitive tasks. This subscale includes alternating symbols and a number of alternating subtests. Divided attention is the ability of an individual to respond appropriately to multiple tasks simultaneously. This subscale includes numerical and monophonic as well as pattern and monophonic detection subtests.

In terms of reliability, the scale scores were between 0.77 and 0.83 for the Cronbach α reliability coefficient, showing its good internal consistency. The test–retest reliability after four weeks was between 0.71 and 0.91. In terms of validity, the correlation between the full scale and each subtest was between 0.63 and 0.77 [29].

### 2.5. Statistical Analyses

Descriptive data of the measured variables are presented as mean and standard deviation (SD) or median and interquartile range (IQR, 25%–75% percentiles). The normality of study variables was examined with the Kolmogorov–Smirnov test. One-way analysis of variation (ANOVA) was used to analyze physical characteristics among the groups. A nonparametric test was used to compare all variables of the ASESC test based on the normality examination. The Kruskal–Wallis H test was used for between-group comparisons, whereas the Wilcoxon signed-rank test was used for within-group comparisons. Significant differences between the means or medians were set as *p* < 0.05. Additionally, Cohen’s d effect size (ES) was used to quantify the magnitude of the training effect. The level of ES was defined as trivial (0.0–0.2), small (0.2–0.6), moderate (0.6–1.2), large (1.2–2.0), and very large (>2.0) [32]. Statistical analyses were conducted using SPSS^®^ Statistics version 25.0 (IBM, Armonk, NY, USA) and Microsoft Excel 2016 (Microsoft Corporation, Redmond, WA, USA).

## 3. Results

### 3.1. Physical Characteristics

The physical characteristics of age, height, and body weight in all study groups are shown in Table 1.

### 3.2. Attention Scales for Elementary School Children Test

As shown in Table 2, qualitative data of each ASESC item are analyzed with the Kruskal–Wallis H test for intergroup comparison and the Wilcoxon signed-rank test for intra-group comparison. For group comparisons, a significant difference was found in item 4 (*p* < 0.019), item 6 (*p* = 0.038), item 9 (*p* = 0.019), and item 10 (*p* = 0.038) of baseline assessment. In the post-training assessment, a significant difference was found in item 1 (*p* < 0.001), item 5 (*p* < 0.001), item 6 (*p* < 0.001), and item 7 (*p* = 0.019). Significant differences of pairwise comparisons between baseline and post-training assessments were identified in items 1, 2, 3, 4, and 5 for the training boys, items 1, 3, 4, 5, 6, and 7 for the training girls, items 3, 4, 6, and 9 for the control boys, and items 6 and 7 for the control girls (*p* < 0.005).

**Figure 2:**
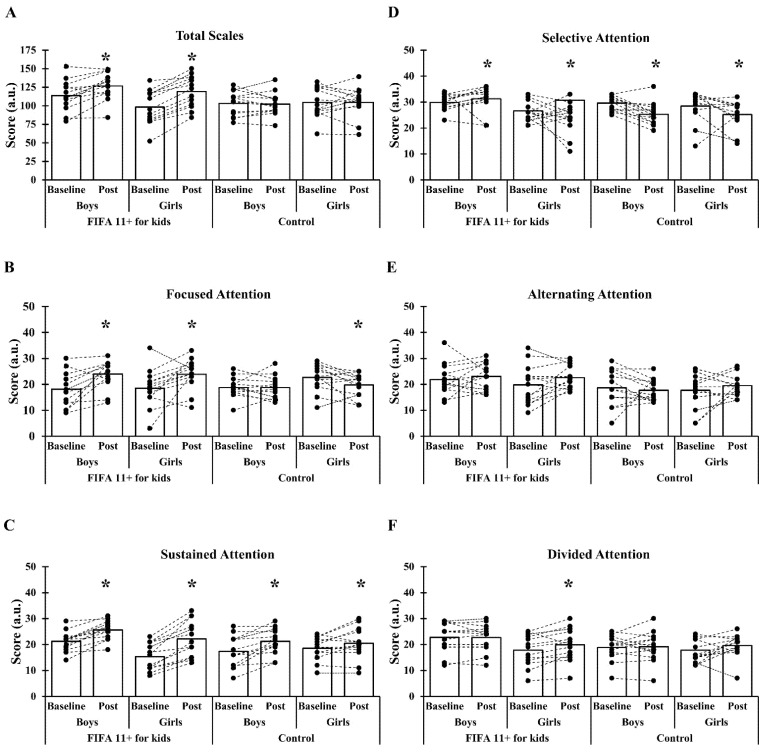
Pooled and individual subscales of the Attention Scale for Elementary School Children test before and after the interventional period in the FIFA 11^+^ for Kids boys, FIFA 11^+^ for Kids girls, control boys, and control girls; (**A**) total scale, (**B**) focused attention, (**C**) sustained attention, (**D**) selective attention, (**E**) alternating attention, and (**F**) divided attention. * indicates significant difference in the Wilcoxon test.

In the total and subscales of the ASESC test (Figure 2), the Kruskal–Wallis H test demonstrates a significant difference in total scale, focused attention, selective attention, and alternating attention (*p* < 0.001). In comparing baseline and post-training assessments (the Wilcoxon signed-rank test), significant differences in pairwise comparison were found in total scale [*p* < 0.001, mean difference (MD) = −12.77, ES = 0.63], focused attention (*p* < 0.001, MD = −5.85, ES = −0.93), sustained attention (*p* < 0.001, MD = −4.38, ES = −1.14), and selective attention (*p* = 0.019, MD = −1.38, ES = −0.33) for the training boys; total scale (*p* < 0.001, MD = −17.15, ES = −0.90), focused attention (*p* = 0.038, MD = −5.15, ES = −0.96), sustained attention (*p* < 0.001, MD = −0.92, ES = −0.15), selective attention (*p* < 0.001, MD = −5.38, ES = −1.29), and divided attention (*p* < 0.001, MD = −0.77, ES = −0.12) for the training girls; sustained attention (*p* < 0.001, MD = −3.85, ES = −0.68) and selective attention for the control boys (*p* < 0.001, MD = 4.31, ES = 1.16); and focused attention (*p* < 0.001, MD = 2.92, ES = 0.58), sustain attention (*p* = 0.019, MD = −1.92, ES = −0.35), and selective attention (*p* = 0.019, MD = 3.31, ES = 0.56) for the control girls.

## 4. Discussion

As its first experimental initiative, the current study was designed to compare gender differences in attentional performances after an 8-week FIFA 11^+^ for Kids training intervention in elementary school children. Participants were assigned to two training groups who participated in the FIFA 11^+^ for Kids intervention and weekly PE lessons, and to two control groups who participated solely in weekly PE lessons. To achieve our research purpose, all children were invited to perform the ASESC test before and after the eight-week study period [29].

It is interesting to note that our results show significant increases in total scale, focused attention, sustained attention, and selective attention in both training groups, and divided attention solely in training girls. This finding demonstrated the positive effects of an 8-week structured exercise program on psychophysiological functions in processing focus-related cues in training children. The benefits of supplementary activities via the FIFA 11^+^ for Kids intervention on attentional capacities could be explained following the “*cardiovascular fitness hypothesis*” [33]. Accordingly, increased cardiovascular fitness, caused by regular physical activity adopted by an individual over time (i.e., longitudinal physical activity program over several weeks) is thought to improve angiogenesis [34] and neurogenesis [35] in areas of the brain that support memory and learning, subsequently enhancing cognitive performance [13,36]. As attention is a central mediator of cognition and learning performance [37,38], it is legitimate to suppose that an individual’s attention capacities can be enhanced by participating in a supplementary chronic physical activity intervention (e.g., the FIFA 11^+^ for Kids program in the present study). Hence, the positive effects of an additional school-based program on attentional performances, as observed in the training boys and girls, could also be explained as following the “*cognitive stimulation hypothesis*” [39,40]. Indeed, the FIFA 11^+^ for Kids intervention could be classified as a cognitively engaging physical activity [28], as most exercises required attention, anticipation, and spatial orientation, particularly while engaged in dual-tasks [26]. Recently, some researchers have argued that chronic physical activities with a relatively high cognitive engagement (where children have to plan strategically and focus attention) have a larger effect on cognitive functions (including attentional capacity, problem solving, etc.) compared to simple physical activities intended to improve cardiovascular performance [20,28,41].

The second hypothesis in the present study assumed that training girls would benefit more from attentional improvement than training boys after the interventional period. This hypothesis could not be determined in focused, sustained, selective, and alternating attentions but it was identified in divided attention. Notably, we used numerical and monophonic, and pattern and monophonic detection subtests to evaluate divided attention in the present study. The children had to identify the right cues to achieve their tasks. It is interesting to note that divided attention is a type of simultaneous attention that allows an individual to synchronize different information cues and successfully carry out multiple tasks in the same period of time [42]. This evidence was supported by the poor capacity for divided attention observed in school children with ADHD [9]. Superior training adaptation regarding divided attention observed in girls could be related to gender differences in brain anatomy. It is well known that the female brain has a higher proportion of gray matter (densely packed with cell bodies), while the male brain has a higher proportion of white matter (consists of myelinated axons that form the connections between brain cells) in the prefrontal cortex [43,44]. In this context, Kanai and Rees [45] highlighted the important role of gray matter in attention. In fact, having more gray matter may explain why young women are usually more efficient at processing information, and usually excel at juggling several activities [46]. As a result, training girls profit more than boys from their participation in continual physical activity in terms of divided attention. It seems that long-term facilitation of the FIFA 11^+^ for Kids intervention could enhance attentional capacity in relation to multiple motor performance tasks in school children. This speculation was supported by the obtained results regarding divided attention in training and/or control girls groups.

Coincidently, participation in both control boys and girls significantly improved sustained attention. This finding indicated an increase in maintaining continuous and repetitive engagement. A possible explanation for this observation is related to daily routine regarding study activities and weekly engagements in school-based PE lessons after the winter vocation in the control groups. A review article conducted de Greeff et al. [36] reported that regular physical activities contribute positively to working memory, sustained attention, and academic performance in preadolescent children. As such, heathy children could possibly benefit their own sustained attention via regular school activities.

Several limitations of this study should be kept in mind, when interpreting the results. First, the small sample size may be a factor for the generalization of our results. This limitation is unavoidable because this investigation was carried out during the first wave of the COVID-19 pandemic in 2020, making it difficult for us to use a larger sample pool. Second, as a result of the same recruitment difficulties, we did not use a control group (inactive) in our experimental procedure. Third, the daily physical activities of both groups were not monitored during the study period. The lack of individual profiles of physical activities may potentially limit the interpretation of our research outcomes. Fourth, although the training children performed exercises according to the FIFA 11^+^ for Kids guidelines, individual variation in training intensity and involvement of group activities may be essential factors affecting training adaptation. Future studies should use tools to quantify training intensity (e.g., heart rate monitor or rating of perceived exertion). Lastly, the level of sexual maturation could be a potential factor influencing the attentional performance between boys and girls. Our findings were limited by the absence of biological examination to exclude the effects of age.

In the present study, attentional capacities were evaluated through convenient measurements. Further research is needed to explore brain adaptation regarding the positive effect of an 8-week FIFA 11^+^ for Kids intervention on attention in elementary school children. This can be performed through empirical measures, such as EEG or functional near-infrared spectroscopy (fNIRS).

## 5. Conclusions

In conclusion, the FIFA 11^+^ for Kids intervention is an effective school-based exercise for attentional improvement in schoolboys and girls. Facilitating an eight-week training program during the semester contributes to optimal performance in focused attention, sustained attention, and selective attention in year 5 schoolboys and girls. Likewise, schoolgirls show positive outcomes in divided attention after a supplementary exercise intervention on school days. The efficiency of the FIFA 11^+^ for Kids intervention for attentional adaptation in association with academic performance and psychometric health needs to be examined in future studies.

## Figures and Tables

**Table 1:** Physical characteristics of the participants.

		FIFA 11^+^ for Kids Boys (*n* = 13)	FIFA 11^+^ for Kids Girls (*n* = 13)	Control Boys (*n* = 13)	Control Girls (*n* = 13)	*p*-Value
Age (years)	Min	11.1	10.9	10.9	10.7	
	Max	11.7	11.6	11.7	11.7	
	Mean ± SD	11.4 ± 0.2	11.3 ± 0.2	11.3 ± 0.2	11.2 ± 0.4	*p* = 0.234
Height (cm)	Min	135.2	126.3	136.2	132.7	
	Max	162.2	155.7	148.7	154.6	
	Mean ± SD	146.1 ± 8.6	142.9 ± 8	141.2 ± 3.9	142.6 ± 6.6	*p* = 0.346
Weight (kg)	Min	26.4	25.1	28.7	23.3	
	Max	74.8	63.3	56.0	51.9	
	Mean ± SD	46.1 ± 14.4	38 ± 10.2	38 ± 7.5	34.4 ± 8.2	*p* = 0.045

Data are presented as minimum, maximum, and mean and standard deviation (Mean ± SD).

**Table 2:** The Attention Scale for Elementary School Children test items scores before and after the interventional period.

Variables	FIFA 11^+^ for Kids Boys (*n* = 13)				FIFA 11^+^ for Kids Girls (*n* = 13)				Control Boys (*n* = 13)				Control Girls (*n* = 13)				Baseline *p*-Value	Post-Test *p*-Value
	Baseline	Post-Test	MD	ES	Baseline	Post-Test	MD	ES	Baseline	Post-Test	MD	ES	Baseline	Post-Test	MD	ES		
Item 1	10.0 (7.0, 13.5)	14.0 (9.5, 15.0) *	−2.69	−0.64 (−1.45, 0.14	9.0 (7.5, 11.5)	13.0 (11.0, 13.5) *	−3.15	−0.93 (−1.78, −0.14)	9.0 (8.0, 11.0)	9.0 (7.5, 11.0)	0.46	0.20 (−0.56, 0.98)	12.0 (9.5, 14.0)	11.0 (9.0, 13.0)	0.46	0.15 (−0.62, 0.92)	0.154	0.000 ^#^
Item 2	9.0 (6.5, 11.5)	12.0 (10.0, 13.0) *	−3.15	−0.98 (−1.82, 0.18	10.0 (6.5, 12.5)	12.0 (9.5, 14.0)	−2.23	−0.54 (−1.34, 0.23)	9.0 (7.5, 12.0)	10.0 (7.0, 12.0)	−0.46	−0.14 (−0.91, 0.63)	12.0 (9.5, 13.0)	10.0 (7.0, 11.0)	−0.46	−0.14 (−0.91, 0.63)	0.288	0.058
Item 3	10.0 (9.0, 11.0)	12.0 (10.0, 14.0) *	−2.08	−0.69 (−1.50, 0.09	7.0 (3.5, 10.0)	11.0 (6.5, 15.0) *	−4.00	−0.97 (−1.81, −0.17)	9.0 (3.0, 11.5)	11.0 (9.0, 12.0) *	−2.00	−0.51 (−1.30, 0.26)	9.0 (6.5, 11.5)	11.0 (7.5, 12.0)	−2.00	−0.39 (−1.17, 0.38)	0.250	0.423
Item 4	12.0 (10.0, 13.0)	14.0 (12.5, 15.0) *	−2.31	−1.04 (−1.89, 0.23	9.0 (5.0, 11.0)	10.0 (8.0, 14.0) *	−2.77	−0.80 (−1.62, −0.02)	9.0 (8.0, 11.5)	11.0 (9.0, 13.5) *	−1.85	−0.75 (−1.56, 0.03)	9.0 (7.0, 12.0)	9.0 (8.0, 14.0)	−1.85	−0.51 (−1.30, 0.27)	0.019 ^#^	0.077
Item 5	15.0 (14.0, 17.0)	17.0 (16.0, 17.0) *	−0.92	−0.61 (−1.41, 0.16	15.0 (14.0, 17.0)	17.0 (16.0, 17.0) *	−0.92	−0.85 (−1.68, −0.06)	17.0 (15.0, 17.0)	14.0 (12.0, 17.0)	2.31	1.08 (0.27, 1.93)	15.0 (12.0, 17.0)	15.0 (12.5, 17.0)	2.31	0.80 (0.01, 1.62)	0.269	0.000 ^#^
Item 6	15.0 (13.0, 16.0)	16.0 (14.5, 17.5)	−0.46	−0.13 (−0.90, 0.64	11.0 (8.5, 14.0)	15.0 (12.0, 16.5) *	−3.15	−0.86 (−1.69, −0.07	14.0 (11.0, 15.5)	11.0 (10.0, 13.0) *	2.00	0.67 (−0.11, 1.48)	15.0 (14.0, 16.0)	12.0 (9.0, 13.0) *	2.00	0.51 (−0.27, 1.30)	0.038 ^#^	0.000 ^#^
Item 7	10.0 (9.0, 14.0)	14.0 (10.5, 14.5)	−1.77	0.48 (−1.27, 0.29	9.0 (7.0, 12.5)	13.0 (11.0, 15.5) *	−3.23	−1.00 (−1.84, −0.20)	9.0 (7.0, 12.0)	10.0 (8.0, 12.0)	−0.62	−0.21 (−0.98, 0.56)	9.0 (4.0, 11.0)	10.0 (9.5, 13.5) *	−0.62	−0.20 (−0.97, 0.57)	0.712	0.019 ^#^
Item 8	10.0 (8.5, 13.0)	11.0 (8.0, 14.5)	0.54	0.13 (−0.64, 0.90	11.0 (7.0, 12.0)	11.0 (7.0, 13.5)	0.46	0.11 (−0.66, 0.88)	11.0 (7.0, 12.0)	8.0 (6.5, 9.0)	1.62	0.43 (−0.34, 1.22)	11.0 (4.5, 13.0)	9.0 (7.0, 10.0)	1.62	0.44 (−0.33, 1.23)	0.962	0.077
Item 9	12.0 (9.5, 14.0)	13.0 (11.0, 15.5)	−0.85	−0.25 (−1.03, 0.52)	9.0 (6.5, 12.5)	11.0 (7.5, 14.5)	−1.31	−0.33 (−1.11, 0.44)	9.0 (8.0, 11.0)	11.0 (9.5, 13.5) *	−1.62	−0.53 (−1.33, 0.24)	9.0 (7.5, 11.0)	11.0 (8.5, 13.0)	−1.62	−0.55 (−1.35, 0.22)	0.019 ^#^	0.192
Item 10	12.0 (8.0, 13.5)	11.0 (7.0, 12.5)	0.92	0.26 (−0.51, 1.04)	8.0 (6.0, 11.5)	9.0 (6.0, 12.5)	−0.77	−0.21 (−0.98, 0.56)	11.0 (7.5, 12.5)	9.0 (4.0, 11.5)	1.31	0.33 (−0.43, 1.12)	8.0 (5.0, 11.0)	11.0 (5.5, 12.0)	1.31	0.41 (−0.36, 1.20)	0.038 ^#^	0.750

Data are presented as median and interquartile (25–75%). Kruskal–Wallis H test was used for group comparison (significant difference indicated as ^#^); Wilcoxon test was used for baseline and post-training comparison (significant difference indicated as *). *n* = number; MD = mean difference; ES = effect size.

## Data Availability

The data are available upon request to the corresponding author via email.
